# The Early Burn Wound Microbiome, Not Donor‐Site Transfer, Marks Graft Healing Outcome

**DOI:** 10.1111/wrr.70217

**Published:** 2026-10-10

**Authors:** Elad Zvi, Carina Chi, Thinh Sieu Tang, Sriya Bobba, Melissa Neely, Roxanne Chatzis, Johannes S. Kern, Shiva Akbarzadeh, Céline Pattaroni

**Affiliations:** ^1^ Victorian Adult Burns Service Bayside Health (The Alfred) Melbourne Australia; ^2^ Department of Surgery, School of Translational Medicine Monash University Melbourne Australia; ^3^ Department of Immunology, School of Translational Medicine Monash University Melbourne Australia; ^4^ Department of Dermatology Bayside Health (The Alfred) Melbourne Australia; ^5^ SkIIN Discovery Program Bayside Health (The Alfred) and Monash University Melbourne Australia

## Abstract

Healing after split‐skin grafting remains difficult to anticipate in small‐area burns, particularly when wounds show no clinically diagnosed infection. We tested whether the burn wound microbiome before grafting captures early wound states linked to subsequent healing. Twenty‐six adults with mid‐dermal to full‐thickness burns involving < 10% total body surface area underwent longitudinal sampling at surgery, graft check and clinic review. At surgery, swabs were collected from the burn wound before grafting, the split‐skin donor site and anatomically matched uninjured control skin. Across 170 infection‐free samples, microbiome profiling showed that pre‐grafting burn wounds already had reduced microbial diversity and distinct community structure compared with matched control skin. This baseline dysbiosis included depletion of commensal‐associated anaerobes, including *Finegoldia* and *Anaerococcus* and enrichment of opportunistic burn‐associated taxa, including *Pseudomonas* and *Acinetobacter*. Donor sites acquired a related wound‐like microbiome and remodelled in parallel with burn wounds during healing, but post‐grafting burn microbiota were not preferentially similar to matched donor sites, arguing against a donor‐to‐burn microbial transfer. The clearest clinical signal was present before grafting: lower burn wound diversity at surgery was associated with delayed healing, and baseline community structure differed in wounds that later required regrafting. These findings suggest that the pre‐grafting burn wound microbiome may provide an early, infection‐independent marker of healing trajectory and should be tested as a predictive biomarker in larger prospective cohorts.

## 1 Introduction

Burn injury remains a major global public health problem, accounting for an estimated 180,000 deaths worldwide annually and contributing substantially to disability‐adjusted life‐years lost among survivors [1]. While modern multidisciplinary burn care has improved survival [2], wound closure and functional recovery remain central clinical challenges. Early excision and split‐skin grafting represent major advances in burn management, reducing mortality and shifting attention towards morbidity, graft take and wound healing outcomes [3]. The pathophysiology of burn injury creates a distinct wound environment. Thermal damage disrupts the skin barrier, exposes nutrient‐rich devitalised tissue and induces local and systemic inflammatory responses [4]. These changes may disturb the normal skin microbiome, a topographically organised ecosystem shaped by local physiology, barrier properties and immune surveillance [5]. In intact skin, microbial communities contribute to barrier defence and immune homeostasis [5]; after injury, this balance can be disrupted, favouring microbial imbalance and expansion of opportunistic taxa even in the absence of clinically diagnosed infection.

Conventional wound swabs and culture‐based diagnostics remain important for identifying suspected pathogens, but they provide limited information about broader microbial community structure, loss of commensal organisms and temporal ecological changes during repair. Sequencing‐based approaches therefore allow burn wounds to be examined as dynamic microbial ecosystems. Few studies have shown that burn injury is associated with microbiome dysbiosis [6, 7, 8] and reduced diversity [8], although findings have been inconsistent and several clinically relevant questions remain unanswered. A key unresolved question is whether early microbial features in burn wounds, before clinically diagnosed infection, are associated with subsequent outcomes such as delayed healing or regrafting. Addressing this question requires distinguishing burn‐associated microbial changes through comparison with anatomically matched uninjured skin. In patients undergoing split‐skin grafting, the donor site provides an additional clinically relevant wound compartment, as it is itself an acute surgical wound and may also contribute microbes to the grafted burn wound.

This study therefore aimed to characterise the perioperative microbiome of small‐area burn wounds requiring split‐skin grafting in the absence of clinically diagnosed infection. By comparing burn wounds with anatomically matched contralateral skin and paired donor sites, the study defines the extent to which burn injury disrupts the normal skin microbiome and whether donor sites acquire a related wound‐like profile. Longitudinal sampling further allowed assessment of microbiome remodelling during healing, the possibility of donor‐to‐burn microbial transfer after grafting and whether early burn wound microbiome features were linked to delayed healing or regrafting.

## 2 Materials and Methods

### 2.1 Patients Recruitment

Adult patients aged 18 years or older with mid‐dermal to full‐thickness burns involving less than 10% total body surface area and requiring grafting were eligible for inclusion. Twenty‐six patients were identified from those admitted to the Victorian Adult Burns Service, Melbourne, Australia, between January 2025 to July 2025. Wounds were reviewed by the investigators to determine suitability for inclusion. All patients received the same institutional perioperative treatment protocol. At grafting, patients received 1–2 g of intravenous cefazolin as surgical antimicrobial prophylaxis. Following grafting, all burn‐wound skin grafts were dressed with Bactigras (chlorhexidine acetate 0.5% w/w in paraffin‐impregnated gauze), and all donor sites were dressed with Xeroform (bismuth tribromophenate 3% in petrolatum‐impregnated gauze). Both dressings were applied intraoperatively, and the same site‐specific dressing protocols were maintained throughout follow‐up until complete wound healing. Thereafter, only non‐antimicrobial topical moisturisers were used. One patient received topical silver sulfadiazine on the burn wound before surgery. Ethical approval was granted by the Alfred Hospital Ethics Committee on 20 December 2024 (project number 805/24).

### 2.2 Sampling

Samples were collected using eSwabs (COPAN Diagnostics). Burn wounds and donor sites were sampled longitudinally at three time points, while anatomically matched control skin was sampled at T1 only. At T1, on the day of surgery, swabs were collected from the burn wound requiring grafting, the donor‐site wound bed immediately after split‐thickness skin graft harvest and unaffected control skin from the same anatomical region as the burn. The burn wound and control skin were sampled before surgical skin preparation. The intact donor‐site epidermis underwent standard antiseptic preparation as part of the sterile field and was then removed for use as the graft. The donor‐site swab was collected from the newly exposed dermal wound bed. The control sample provided a within‐patient, anatomically matched reference for identifying burn‐associated microbiome differences at surgery. At T2, on postoperative day 3–4 during graft assessment, the burn wound and donor site were sampled after the dressings were opened and before cleaning, dressing changes, or other clinical interventions. At T3, during outpatient assessment 1–4 weeks after surgery, both wound sites were again sampled before cleaning, dressing changes, or other clinical interventions.

### 2.3 DNA Extraction and 16S rRNA Gene Sequencing

Skin swabs were collected using eSwabs (COPAN Diagnostics) and media were centrifuged at 14,000 g for 10 min at 4°C. Negative controls included eSwab, extraction and PCR. Microbial DNA was extracted as previously described [9] using the DNeasy UltraClean Microbial Kit (Qiagen). Bacterial community profiling was performed by amplification of the V1–V2 region of the 16S rRNA gene using custom barcoded primers based on the F27/R338 primer pair:

**16S‐Forward:**

5′‐AATGATACGGCGACCACCGAGATCTACACTATGGTAATTCCAGMGTTYGATYMTGGCTCAG‐3′.

**16S‐Reverse:**

5′‐CAAGCAGAAGACGGCATACGAGATACGAGACTGATTNNNNNNNNNNNNAAGCTGCCTCCCGTAGGAGT‐3′.

PCR reaction consisted of microbial DNA‐free water, 1 μL of each primer at 5 μM, 2.5 μL of Accuprime PCR buffer II (Thermo Fisher Scientific), 10 μL of DNA template and 1 μL of Accuprime Taq polymerase (Thermo Fisher Scientific) with initial denaturation for 3 min at 94°C, followed by 35 cycles (16S) of 30‐s denaturation at 94°C, 30‐s annealing at 56°C (16S) and 60‐s elongation at 68°C, with a final extension at 68°C for 10 min. Amplicons were then pooled in equimolar amounts, purified using the AMPure XP bead cleanup system and sequenced on an Illumina MiSeq platform using a 500‐cycle kit.

### 2.4 Sequence Processing and Quality Control

Processing of the raw sequencing data was executed through the microbiome‐dada2 workflow [10] using the dada2 R package [11]. Chimera‐filtered amplicon sequence variant (ASV) count tables, taxonomic assignments and sample metadata were imported using the phyloseq R package [12]. ASVs lacking family‐level classification were removed, together with the known contaminant genus *Brevibacillus*. Contaminants were further identified using prevalence‐based filtering with the decontam R package [13] against negative controls, and features detected in at least two negative controls were excluded. Samples with fewer than 2000 reads, as well as positive controls, were removed prior to downstream analyses. A phylogenetic tree was reconstructed from ASV sequences following multiple sequence alignment with DECIPHER [14] and maximum‐likelihood tree inference with phangorn [15]. Closely related taxa were then agglomerated phylogenetically and subsequently collapsed at the species level where appropriate, and taxa were further filtered to retain features with counts greater than 1 in more than 20% of samples. For downstream ordination, each comparison‐specific subset was normalised independently using cumulative sum scaling in metagenomeSeq [16].

### 2.5 Statistical Analysis

Alpha diversity was assessed using the Shannon index. Paired Wilcoxon tests were used for comparisons between burn wounds and matched control skin. Temporal comparisons within burn wounds and donor sites were assessed using pairwise Wilcoxon tests with false‐discovery‐rate adjustment, and between‐group comparisons within individual timepoints were performed using Wilcoxon rank‐sum tests. Beta diversity was assessed using Bray–Curtis dissimilarities, visualised by principal coordinates analysis and tested using permutational multivariate analysis of variance with adonis2 (vegan R package [17]). Differential abundance analysis was performed at ASV level using linear models for differential abundance analysis of microbiome compositional data (LinDA [18]) with a significance threshold of 0.05. Patient was included as a model term where appropriate for paired or repeated‐measures designs. Significantly differentially abundant taxa were summarised as log2 fold‐change bar plots.

## 3 Results

### 3.1 Study Design

Twenty‐six adults with mid‐dermal to full‐thickness burns involving less than 10% total body surface area (TBSA) and requiring split‐skin graft surgery were recruited. For each participant, swabs were collected at the time of surgery (T1) from the burn wound, the split‐skin donor site and an anatomically matched uninjured control site. Follow‐up swabs were then collected from the burn wound and donor site at graft check (T2, approximately 4 days after surgery) and again at clinic review (T3, approximately 25 days after discharge) (Figure 1a). To minimise confounding by infection and capture the natural temporal dynamics of wound healing, only samples collected in the absence of a clinically diagnosed infection were included in the analysis. Microbiome composition was characterised by 16S rRNA gene sequencing. After quality control, 170 samples from the 26 participants were retained: 74 burn‐wound samples, 70 donor‐site samples and 26 matched control samples. Because longitudinal samples were not available or did not pass sequencing quality‐control thresholds at every timepoint, the number of analysable samples varied between outcome analyses. Long‐term healing outcomes, including delayed healing and other complications, were assessed at T3. Five patients had delayed healing and three required regrafting.

**FIGURE 1:**
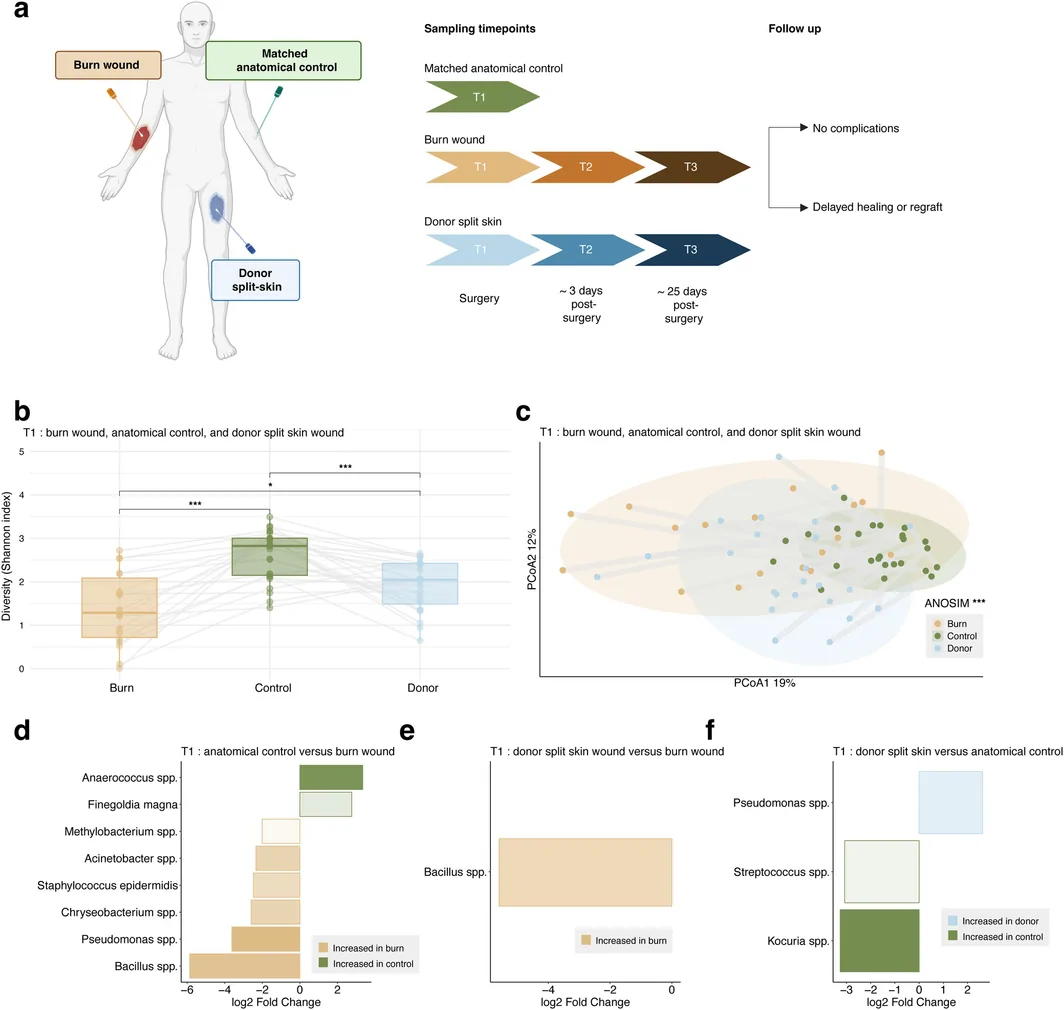
Baseline perioperative microbiome features of burn wounds, donor split‐skin wounds and matched anatomical control skin. (a) Study design and sampling scheme showing collection of burn wound, donor split‐skin wound and anatomically matched contralateral control swabs at surgery (T1), with follow‐up sampling of burn and donor wounds at graft check (T2) and clinic review (T3). (b) Shannon diversity at T1 across burn wounds, matched anatomical control skin and donor split‐skin wounds. (c) Principal coordinates analysis (PCoA) of Bray–Curtis distances at T1. (d) Differentially abundant taxa between burn wounds and matched anatomical control skin at T1 identified using LinDA. Differentially abundant taxa between burn wounds and donor split‐skin wounds at T1 identified using LinDA. (e) Differentially abundant taxa between burn wounds and donor split‐skin wounds at T1 identified using LinDA. (f) Differentially abundant taxa between donor split‐skin wounds and matched anatomical control skin at T1 identified using LinDA. Grey lines connect matched samples from the same patient. Differentially abundant taxa are shown as significant log2 fold changes only. *p < 0.05, **p < 0.01, ***p < 0.001; ns, not significant.

### 3.2 Burn Wounds Harbour a Distinct Microbiome Compared With Matched Contralateral Control Skin, While Donor Sites Display a Wound‐Like Microbial Profile

To define how burn injury alters the skin microbiome prior to surgical intervention, we compared burn wound samples collected at surgery (T1) with anatomically matched uninjured control skin from the corresponding contralateral site, providing a patient‐specific reference for the expected baseline skin microbiome and with donor split‐skin wound samples collected at the same timepoint. Shannon diversity was significantly lower in burn wounds than in anatomically matched control skin at T1 (Wilcoxon rank‐sum exact test, FDR‐adjusted *p* < 0.001), while donor‐site samples showed values intermediate between burn wounds (Wilcoxon rank‐sum exact test, FDR‐adjusted *p* = 0.042) and controls (Wilcoxon rank‐sum exact test, FDR‐adjusted *p* < 0.001) (Figure 1b). Similarly, overall microbial community composition significantly differed across sample types at surgery (Bray–Curtis PERMANOVA, burn: *R* ^2^ = 0.108, *p*‐value < 0.001), with the clearest separation observed between burn wounds and matched anatomical control skin, whereas donor‐site communities occupied a partially overlapping, intermediate position (Figure 1c). Differential abundance analysis showed that most compositional differences were between burn wounds and matched control skin at T1 (Figure 1d). Burn wounds were enriched for *Bacillus* spp., *Pseudomonas* spp., *Staphylococcus epidermidis* and *Acinetobacter* spp., whereas *Anaerococcus* spp. and *Finegoldia magna* were enriched in matched control skin. Comparison of burn wounds with donor split‐skin wounds identified *Bacillus* spp. as the only differentially abundant feature, with higher abundance in burn wounds (Figure 1e). In contrast, donor sites differed from anatomical control skin by enrichment of *Pseudomonas* spp. in donor samples, whereas *Streptococcus* spp. and *Kocuria* spp. were higher in control skin (Figure 1f). Thus, the donor site microbiome was more similar to the burn wound than to matched uninjured skin, consistent with a shared wound‐associated microbial shift. Notably, *Pseudomonas* spp. was increased in both burn and donor sites relative to control skin, whereas *Bacillus* spp. appeared more specifically associated with burn wounds.

### 3.3 Burn Wounds and Split‐Skin Donor Sites Undergo Parallel Temporal Microbiome Remodelling During Healing, Without Clear Evidence of Donor‐Site Microbiome Transfer

We next examined temporal microbiome remodelling in burn wounds and split‐skin donor sites during healing (Figure 2). Overall microbial community composition differed significantly across timepoints in both burn wounds (Figure 2a) and split‐skin donor sites (Figure 2b) (Bray–Curtis PERMANOVA, burn: *R* ^2^ = 0.072, *p*‐value < 0.001; donor: *R* ^2^ = 0.095, *p*‐value < 0.001). Alpha diversity did not differ significantly across timepoints in either burn wounds (Figure 2c) or split‐skin donor sites (Figure 2d), although donor sites showed a trend towards increased diversity between T2 and T3 (Wilcoxon rank‐sum exact test, FDR‐adjusted *p* = 0.079). Differential abundance analysis identified six temporally changing ASVs in each tissue (Figure 2e,f). Shared temporal shifts were observed in both burn wounds and split‐skin donor sites, including increased abundance of *Micrococcus* spp. and *Micrococcus luteus* at later timepoints and decreased abundance of *Enterococcus* spp. over time. Because donor skin is grafted onto the burn wound, we next asked whether the donor‐site microbiome might contribute directly to the post‐grafting burn microbiome. To test this, we compared T1 donor‐site samples, expected to most closely reflect the microbiome present at the time of graft transfer, with T2 burn‐wound samples collected after grafting (Bray–Curtis PERMANOVA, burn: *R* ^2^ = 0.052, *p*‐value < 0.01) (Figure 2g). We then asked whether each T2 burn sample was more similar to its paired T1 donor‐site sample from the same patient than to donor‐site samples from other patients. This was not the case (Figure 2h), arguing against a patient‐specific donor‐to‐burn transfer signal. Together, these findings show that both burn wounds and split‐skin donor sites undergo parallel temporal microbiome remodelling during healing, with overlapping differentially abundant ASVs. They also suggest that the similarity between donor and burn wound microbiota is more consistent with a shared wound‐associated remodelling process than with direct microbial transfer from the donor site to the grafted burn wound.

**FIGURE 2:**
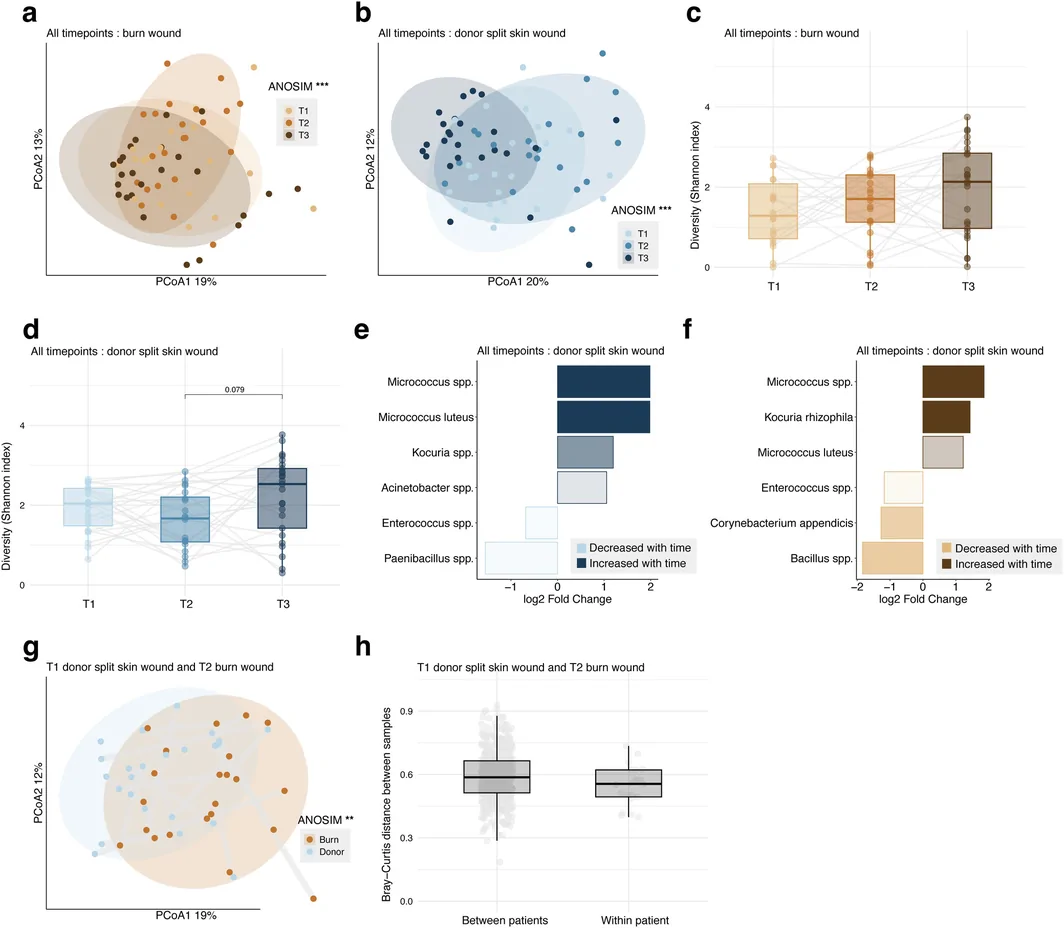
Burn wounds and donor split‐skin wounds undergo parallel temporal microbiome remodelling during healing. (a, b) Principal coordinates analysis (PCoA) of Bray–Curtis distances across timepoints in burn wounds and donor split‐skin wounds, respectively. (c, d) Shannon diversity across timepoints in burn wounds and donor split‐skin wounds, respectively. (e, f) Differentially abundant ASVs across time in burn wounds and donor split‐skin wounds, respectively, identified using LinDA. (g) PCoA of Bray–Curtis distances comparing T1 donor split‐skin wound samples with T2 burn wound samples after grafting. (h) Bray–Curtis distance between T2 burn wounds and T1 donor split‐skin wounds for within‐patient matched pairs versus between‐patient comparisons. Grey lines connect longitudinal samples or matched donor–burn pairs from the same patient. Differentially abundant ASVs are shown as significant log2 fold changes only. *p < 0.05, **p < 0.01, ***p < 0.001; ns, not significant.

### 3.4 Early Burn Wound Microbiome Differences Are Associated With Delayed Healing and Regrafting Outcome

We next examined whether burn wound microbiome features across follow‐up were associated with subsequent healing outcome (Figure 3). At surgery (T1), overall microbial community composition did not differ significantly between wounds that later showed delayed healing and those with no complications (Bray–Curtis PERMANOVA, *R* ^2^ = 0.092, *p*‐value = 0.221) (Figure 3a). However, Shannon diversity was significantly lower in burn wounds that subsequently showed delayed healing (Wilcoxon rank‐sum test, *p*‐value < 0.05) at that timepoint (Figure 3b). At graft check (T2), the clearest difference was observed at the community level, with microbial composition differing significantly by healing outcome (Bray–Curtis PERMANOVA, *R* ^2^ = 0.110, *p*‐value < 0.05) (Figure 3c). Shannon diversity at T2 showed a trend towards lower values in delayed‐healing wounds, although this difference was not statistically significant (Wilcoxon rank‐sum test, *p* = 0.143) (Figure 3d). By clinic review (T3), no significant differences between healing outcome groups were detected in either overall microbial community composition (Bray–Curtis PERMANOVA, *R* ^2^ = 0.057, *p*‐value = 0.483) (Figure 3e) or Shannon diversity (Wilcoxon rank‐sum test, *p*‐value = 0.075) (Figure 3f). Patients with delayed healing did not differ significantly from those without complications in time from injury to grafting (Wilcoxon rank‐sum test, *W* = 19, FDR‐adjusted *p*‐value = 0.587) and hospital stay (*W* = 15, FDR‐adjusted *p*‐value = 0.094) (Supplementary Figure 1a), or systemic antibiotic administration at sampling (Fisher's exact test, FDR‐adjusted *p*‐value = 1.000) (Supplementary Figure 1b). Together, these findings indicate that microbiome differences associated with delayed healing were most evident early, with lower alpha diversity at T1 and altered community composition at T2, independently of time from injury to grafting, hospital stay and systemic antibiotic administration at sampling. We next examined whether wounds that subsequently required regrafting showed distinct microbiome features before regrafting, and whether these differences were still detectable after regrafting. At surgery (T1), overall microbial community composition differed significantly between wounds that later required regrafting and wounds with no complications (Bray–Curtis PERMANOVA, *R* ^2^ = 0.257, *p*‐value < 0.01) (Figure 3g). No significant differences in Shannon diversity were observed between groups at T1 (Wilcoxon rank‐sum test, *p* = 0.365) (Figure 3h). At the post‐regraft sampling timepoint, representing a new surgical baseline for regrafted wounds, no significant difference was detected in either overall microbial community composition (Bray–Curtis PERMANOVA, *R* ^2^ = 0.057, *p*‐value = 0.587) (Figure 3i) or Shannon diversity (Wilcoxon rank‐sum test, *p* = 0.146) (Figure 3j). Patients requiring regrafting had longer hospital stays than those without complications (Wilcoxon rank‐sum test, *W* = 0, FDR‐adjusted *p*‐value = 0.032) (Supplementary Figure 1a). All three patients requiring regrafting were also receiving systemic antibiotics at sampling, compared with 2/13 patients without complications (Supplementary Figure 1b). Together, these findings indicate that the microbiome differences observed before regrafting were no longer detectable after regrafting, with post‐regraft wounds showing a profile comparable to wounds with no complications.

**FIGURE 3:**
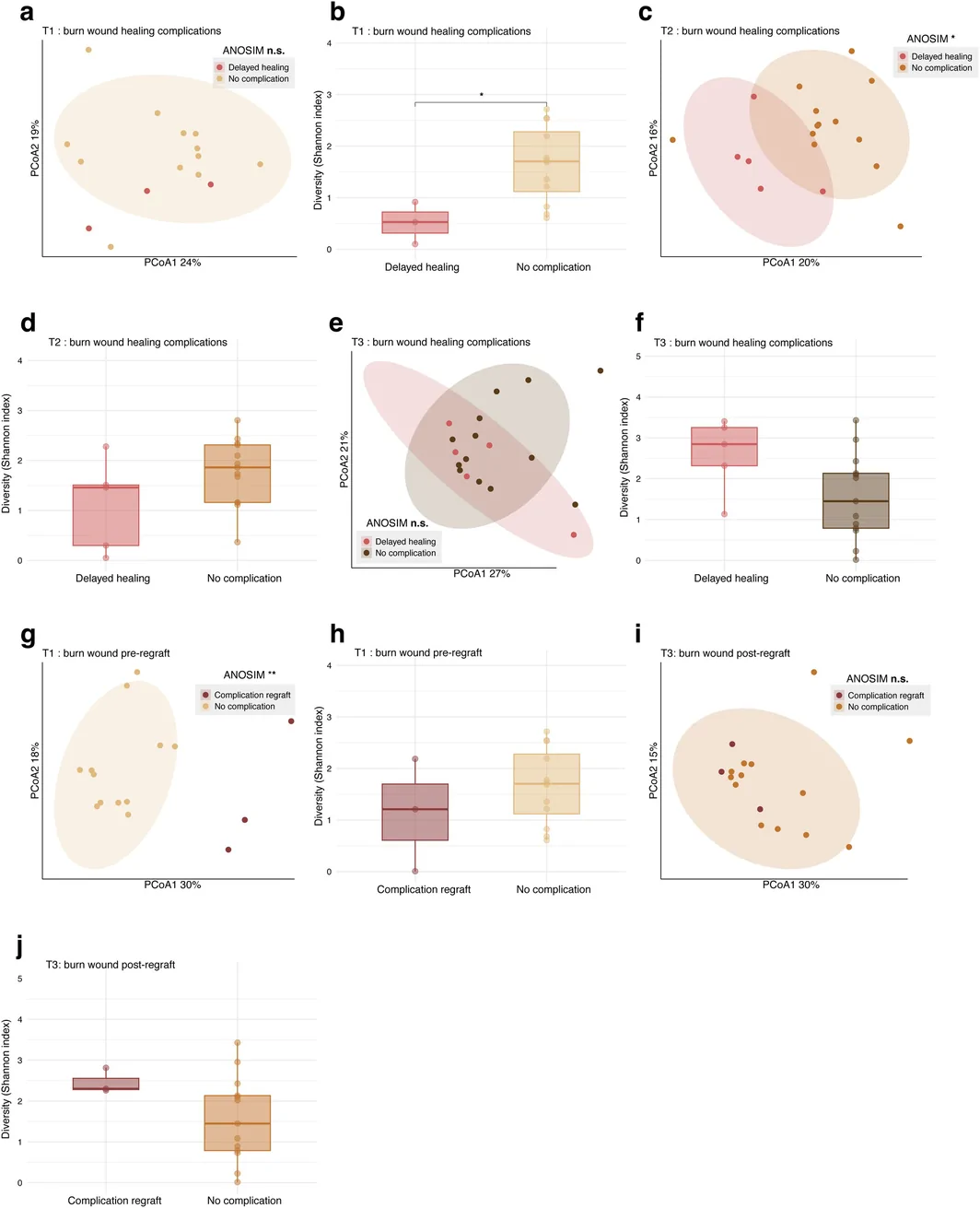
Early burn wound microbiome features are associated with subsequent healing outcome. (a) Principal coordinates analysis (PCoA) of Bray–Curtis distances in burn wounds at T1, stratified by delayed healing versus no complication. (b) Shannon diversity in burn wounds at T1 by healing outcome. (c) PCoA of Bray–Curtis distances in burn wounds at T2, stratified by delayed healing versus no complication. (d) Shannon diversity in burn wounds at T2 by healing outcome. (e) PCoA of Bray–Curtis distances in burn wounds at T3, stratified by delayed healing versus no complication. (f) Shannon diversity in burn wounds at T3 by healing outcome. (g) PCoA of Bray–Curtis distances at T1 comparing burn wounds that later required regrafting with wounds that healed without complications. (h) Shannon diversity at T1 for the corresponding regraft comparison. (i) PCoA of Bray–Curtis distances at T3 comparing post‐regraft burn wounds with wounds that healed without complications. (j) Shannon diversity at T3 for the corresponding regraft comparison. Points represent individual samples and ellipses indicate group dispersion. *p < 0.05, **p < 0.01, ***p < 0.001; ns, not significant.

## 4 Discussion

The study aimed to compare burn and donor‐site microbiomes with anatomically matched control skin at surgery, characterise changes during healing and assess associations with healing outcomes. Using anatomically matched contralateral skin as a patient‐specific reference, burn wounds at the time of surgery displayed a distinct microbiome characterised by reduced alpha diversity and altered community composition. The reduction in alpha diversity is consistent with previous work [8] showing lower diversity in burn wounds than in spared skin during the acute phase. Inclusion of donor‐site samples extended this comparison to a second acute wound compartment. Donor‐site communities closely resembled burn wounds, with only a single differentially abundant ASV. However, burn wounds showed lower alpha diversity and a larger number of differentially abundant ASVs relative to control skin, indicating a more extensive microbiome disruption. Together, these findings suggest that acute skin injury is associated with the emergence of a wound‐like microbiome, while the burn wound retains the most pronounced microbial alterations. At the taxonomic level, early burn dysbiosis was marked by depletion of *Finegoldia magna* and *Anaerococcus* and enrichment of *Bacillus*, *Pseudomonas*, *Staphylococcus epidermidis* and *Acinetobacter*. *Finegoldia* and *Anaerococcus* are both Gram‐positive anaerobic cocci that form part of the normal skin microbiota. Interestingly, experimental work has shown that these commensals can directly stimulate keratinocyte host‐defence programs, including antimicrobial peptide production and inflammatory mediators, supporting the concept that they contribute to skin homeostasis and early barrier defence [19]. Their depletion in burn wounds may therefore reflect loss of beneficial host–microbe interactions following thermal injury. By contrast, enrichment of *Pseudomonas* and *Acinetobacter* in burn wounds sampled in the absence of clinically diagnosed infection is clinically relevant, as both genera include major burn‐associated opportunists commonly implicated in hospital‐acquired and often antimicrobial‐resistant wound infections [20]. Inclusion of the donor site refined this pattern further. *Pseudomonas* was increased in both burn and donor sites relative to control skin, suggesting that its expansion may represent a broader feature of the early wound environment rather than a burn‐specific signature. In contrast, *Bacillus* was the only taxon that distinguished burn wounds from donor sites, with higher abundance in burn wounds, indicating that some aspects of the burn microbiome are more specific to the burn wound itself. This is also clinically relevant, as donor wounds, like burn wounds, are themselves susceptible to infectious complications [21, 22]. Together, these findings suggest that donor sites rapidly acquire a wound‐like microbiome that overlaps substantially with burn wounds, although the burn wound retains the most pronounced microbial alterations.

A second key finding was that burn wounds and split‐skin donor sites underwent parallel temporal microbiome remodelling. Although alpha diversity did not change significantly over time in either tissue, community composition shifted significantly in both, with several shared changes, including an increase in *Micrococcus* spp. and *Micrococcus luteus* and a decrease in Enterococcus spp. at later timepoints. This suggests that part of the microbial succession observed during recovery reflects a general response to acute skin injury and repair rather than a process unique to burn wounds. Both burn wounds and donor sites are characterised by barrier disruption, inflammation, re‐epithelialisation and exposure to perioperative wound care, all of which are likely to impose overlapping ecological pressures on the microbiota. All patients received standardised perioperative antimicrobial prophylaxis and site‐specific postoperative dressing protocols. These shared exposures are therefore unlikely to explain differences in microbiome composition or healing outcomes between patients. However, Bactigras and Xeroform both contain antimicrobial compounds and may have influenced longitudinal microbiome remodelling at the burn and donor sites. The observed temporal changes therefore represent wound healing under standardised antimicrobial dressing protocols. We also considered whether donor skin might directly contribute microbiota to the burn wound after grafting. Although T1 donor and T2 burn samples showed substantial compositional similarity overall, T2 burn samples were not more similar to their paired T1 donor sample from the same patient than to donor samples from other patients. Together, these findings argue against a strong patient‐specific donor‐to‐burn transfer signal and instead support the interpretation that donor and burn wounds undergo broadly similar, parallel microbiome remodelling during healing.

Finally, early microbiome features were associated with subsequent healing outcomes. At surgery, wounds that later showed delayed healing already had lower alpha diversity, despite the absence of a significant difference in overall community composition at that timepoint. By graft check, the signal shifted from diversity to community structure, with significant compositional differences according to healing outcome. These associations were no longer evident by clinic review, suggesting that microbiome features linked to poor healing may be most informative early, before later wound evolution and clinical management reduce the contrast between healing trajectories. Importantly, these early microbiome differences preceded the healing outcome and were observed despite standardised perioperative prophylaxis and postoperative dressing protocols across the cohort. They were also independent of additional systemic antibiotic administration at sampling, time from injury to grafting and hospital stay. Wounds that later required regrafting also showed distinct microbial community composition at the first surgery timepoint, but these differences were no longer evident after regrafting. However, all three patients requiring regrafting received systemic antibiotics at sampling and had longer hospital stays than patients without complications. Although this subgroup was small, these findings raise the possibility that regrafting resets the wound microbial landscape by creating a new surgical baseline and altering the local ecological environment. Whether this reflects removal of a previously dysbiotic wound bed, restoration of barrier conditions more favourable to recovery, or both, cannot be determined from the present data. Small‐area burns provided a defined clinical setting in which to examine local wound–microbiome associations with less influence from the systemic effects of major burn injury. The early microbial associations identified here require validation in larger or higher‐risk burn cohorts, where delayed healing and graft failure have greater clinical consequences.

Overall, the findings indicate that burn injury is associated with early disruption of the normal skin microbial ecosystem, including reduced diversity, loss of anatomical structuring, depletion of the commensal‐associated anaerobes *Finegoldia* and *Anaerococcus* and enrichment of potential pathogenic microbes such as *Pseudomonas*. Donor sites also rapidly acquired a wound‐like microbiome, and both burn wounds and donor sites underwent partly shared temporal microbiome remodelling during healing, consistent with common ecological pressures associated with acute skin injury and repair. However, the post‐grafting burn microbiome was not detectably closer to the paired donor‐site microbiome from the same patient than to donor microbiomes from other patients, arguing against a strong patient‐specific donor‐to‐burn transfer signal. Most importantly, microbiome differences linked to delayed healing and regrafting were most evident at early timepoints, supporting the view that the initial burn wound microbiome, rather than later remodelling or donor‐site profiles, is likely to be the most informative indicator of subsequent healing trajectory. Larger prospective studies are now needed to determine whether these early burn‐wound microbial features can be translated into clinically useful biomarkers of healing outcome.

## Conflicts of Interest

The authors declare no conflicts of interest.

## Supporting information


**Supplementary Figure 1.** Clinical timing and systemic antibiotic administration by healing outcome. (a) Time from injury to grafting and total hospital stay in patients with no complications, delayed healing, or requiring regrafting. Bars show mean ± SD, and points represent individual patients. Pairwise comparisons were performed using Wilcoxon rank‐sum tests with FDR correction across the three comparisons for each measure. (b) Proportion of patients receiving systemic antibiotics at sampling according to healing outcome. Numbers within bars indicate patient counts. Pairwise comparisons were performed using two‐sided Fisher's exact tests with FDR correction across the three comparisons. *FDR‐adjusted *p*‐value < 0.05; ns, not significant.

## Data Availability

16S rRNA gene sequencing data generated in this study are deposited in the NCBI Sequence Read Archive (SRA) under the Bioproject PRJNA1459613.
