# Supplementary material for: The Early Burn Wound Microbiome, Not Donor‐Site Transfer, Marks Graft Healing Outcome
Source: Wound Repair Regen. 2026 Oct 10;34(5):e70217. doi: 10.1111/wrr.70217 (PMC13653241; doi:10.1111/wrr.70217)
Supplement: Supplementary file 1 [file WRR-34-0-s001.pdf]

Supplementary information

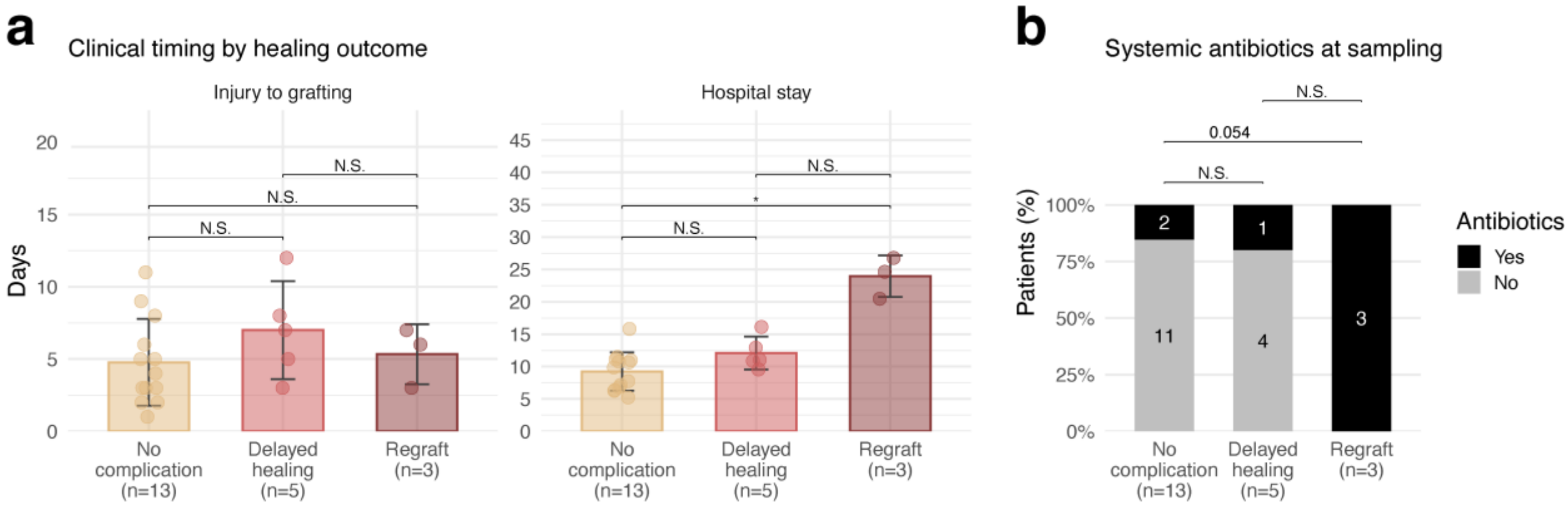

SUPPLEMENTARY FIGURE 1. Clinical timing and systemic antibiotic administration by healing outcome. (**a**) Time from injury to grafting and total hospital stay in patients with no complications, delayed healing, or requiring regrafting. Bars show mean ± SD, and points represent individual patients. Pairwise comparisons were performed using Wilcoxon rank-sum tests with FDR correction across the three comparisons for each measure. (**b**) Proportion of patients receiving systemic antibiotics at sampling according to healing outcome. Numbers within bars indicate patient counts. Pairwise comparisons were performed using two-sided Fisher's exact tests with FDR correction across the three comparisons. *FDR-adjusted P-value < 0.05; n.s., not significant.
